# The Influence of Region, Sex, and Age on the Prevalence of Gastrointestinal Parasites in Alpacas (*Vicugna pacos*) in Poland

**DOI:** 10.3390/ani15060841

**Published:** 2025-03-14

**Authors:** Bogumiła Pilarczyk, Renata Pilarczyk, Marta Juszczak-Czasnojć, Małgorzata Bąkowska, Agnieszka Tomza-Marciniak, Beata Seremak, Paulius Matusevičius, Ramutė Mišeikienė

**Affiliations:** 1 Department of Animal Reproduction Biotechnology and Environmental Hygiene, Faculty of Biotechnology and Animal Husbandry, West Pomeranian University of Technology, 71-270 Szczecin, Poland; bogumila.pilarczyk@zut.edu.pl (B.P.); malgorzata.bakowska@zut.edu.pl (M.B.); agnieszka.tomza-marciniak@zut.edu.pl (A.T.-M.); beata.seremak@zut.edu.pl (B.S.); 2 Laboratory of Biostatistics, Faculty of Biotechnology and Animal Husbandry, West Pomeranian University of Technology, 71-270 Szczecin, Poland; renata.pilarczyk@zut.edu.pl; 3 Department of Animal Nutrition Veterinary Academy, Lithuanian University of Health Sciences, Tilžės 18, LT-47181 Kaunas, Lithuania; paulius.matusevicius@lsmu.lt; 4 Institute of Animal Rearing Technologies, Veterinary Academy, Lithuanian University of Health Sciences, Tilžės 18, LT-47181 Kaunas, Lithuania; ramute.miseikiene@lsmu.lt

**Keywords:** nematodes, protist, *Eimeria* sp., *Nematodirus* spp., *Strongyloides* sp., coccidia, infection

## Abstract

The aim of the study was to determine the extent and intensity of gastrointestinal parasite infections in alpacas (*Vicugna pacos*) in Poland, considering factors such as region, gender, age, and season. The research was conducted on 29 herds located throughout Poland. Faecal samples from 512 alpacas were collected in autumn 2023 and spring 2024. Examination was performed using the direct flotation and McMaster methods. The average prevalence of gastrointestinal parasitic infections was 74.4%; 68.8% of alpacas were infected with nematodes and 34.8% with protists of the genus *Eimeria*. The most commonly detected parasites were *Nematodirus* spp. (38.5%) and *Eimeria punoensis* (18.2%). The analysis revealed significant differences in the intensity of gastrointestinal parasitic infections depending on region, age, gender, and season, suggesting the need to tailor deworming programs to the specific requirements of different groups of alpacas. Furthermore, regional differences in the prevalence of *Strongyloides* sp. and *Strongylida* infections were observed, indicating a significant impact of local environmental conditions and husbandry practices on the risk of infection. Seasonal variations in the intensity of *Eimeria* infections, particularly in autumn, were also noted. Parasitic infections in alpacas in Poland are common and generally asymptomatic. These results highlight the need for the implementation of a comprehensive preventive program for controlling parasitic infection, including regular diagnostics, pasture management optimization, maintenance of hygiene, and selective use of antiparasitic drugs in alpacas.

## Simple Summary

Recent years have seen a steady increase in the number of South American camelids, including alpacas, in Europe. Alpaca breeding programs typically keep the animals in livestock buildings and covered sheds throughout the year to provide protection from adverse weather conditions while in the paddocks. Such farming, especially with poor hygiene, can encourage the accumulation of eggs and oocysts of gastrointestinal parasites, thus favouring the spread of disease in the herd. Gastrointestinal parasites in alpacas are a serious health problem. This study showed that the average prevalence of gastrointestinal parasitic infections was 74.4%. Over 68% of alpacas were infected with nematodes and 34.8% with protists of the genus *Eimeria*. The most commonly detected parasites were *Nematodirus* spp. (38.5%) and *Eimeria punoensis* (18.2%). In addition, animals younger than one year demonstrated a significantly greater intensity of infection with *Nematodirus* spp., *Trichuris* sp., *E. macusaniensis*, *E. lamae*, *E. alpacae,* and *E. punoensis*, as well as combined nematodes and *Eimeria*. Therefore, it is important to regularly test faeces for the presence of parasite eggs and oocysts, rotate pastures, maintain housing hygiene, and perform targeted deworming to help minimise the risk of infection in alpacas.

## 1. Introduction

Recent years have seen a steady increase in the number of South American camelids (SAC), including alpacas, in Europe. The Polish population is estimated to be around 5500 [1], although a precise estimation of their number is difficult, as they have only been bred for a short period, and not all herds are officially registered. Indeed, they were only recognized as livestock in Poland in December 2020. Nevertheless, their numbers are believed to be increasing, which underlines the need for detailed research on the species. A fuller understanding of the health issues associated with alpaca ownership would be of great value for both current and future breeders, as well as for veterinarians, to implement effective treatment and health management strategies in Central European breeding conditions.

Alpaca breeding programs in Poland typically keep the animals in livestock buildings and covered sheds throughout the year to provide protection from adverse weather conditions while in the paddocks. Such conditions, and the resulting stress, can have a significant impact on the prevalence and intensity of gastrointestinal parasite infection. Keeping animals in the same building or pasture all year round, especially with poor hygiene, can encourage the accumulation of eggs and oocysts of gastrointestinal (GI) parasites, thus favouring the spread of disease in the herd. Gastrointestinal parasites in alpacas are a serious health problem [2].

Despite significant progress and an increasing number of farms, there is still a lack of research in Poland on the impact of parasites on the economic performance of farmers and their effects on other animals. However, in other countries, such as Australia, studies have been carried out indicating significant relationships in this area. A study by Windsor et al. [3] showed a statistically significant effect of regular deworming treatments on the health of alpacas and the economic outcomes associated with their breeding. The results indicated that after four months, male alpacas subjected to deworming gained an average of 3.1 kg more body weight and produced 0.36 kg more wool compared to the control group. In the case of dewormed females, a weight gain of 1.9 kg more was observed than in non-dewormed females, although the weight of their wool was slightly lower (by 0.03 kg). However, the introduction of alpacas into the Polish environment is associated with significant epidemiological challenges. Alpacas bring with them a species-specific parasitic fauna, which increases the risk of introducing new parasite species into local ecosystems, potentially threatening endemic biodiversity [4]. At the same time, contact between alpacas and native parasite species in Europe creates conditions for new parasite–host relationships to emerge. Such interactions may have far-reaching epidemiological consequences and require detailed research [5].

The presented research data of Szopieray et al. [6] and Nosal et al. [7] clearly indicate a serious parasitic problem in alpaca farms in Poland. The high prevalence of both nematodes and coccidia, as well as the identification of species with proven pathogenicity, confirms the need for comprehensive preventive measures. A study conducted on six alpaca farms by Szopieray et al. [6] found that 57.7% of alpacas were infected with parasites, with nematodes predominating, including *Nematodirus* sp. (28.9%), *Trichostrongylus* sp. (15.5%), and *Strongyloides* sp. (13.4%). Less common were *Trichuris* sp. (3.1%), *Capillaria* spp. (2.1%), *Oesophagostomum* sp. (1.0%), and eggs of *Moniezia* sp. (1.0%). Oocysts of *Eimeria macusaniensis* were found in 8.2% and other species of the genus *Eimeria* in 4.1%. In contrast, in a study by Nosal et al. [7] on 13 alpaca herds, oocysts of coccidia were present in 31.9% of the animals, with an average infection intensity of 213 OPG (20-5840 OPG). The most frequently detected coccidian species were *Eimeria punoensis* (23.7%) and *E. alpacae* (7.4%), as well as the highly pathogenic species *E. macusaniensis* (3.1%) and *E. lamae* (1.9%). The nematode prevalence in the study by Nosal et al. [7] was 54.1%, with a mean intensity of 106 EPG (20-2060 EPG). Studies carried out in other countries confirm that coccidiosis, caused by *Eimeria* spp., poses a particularly serious threat to young alpacas (cria). It is the main cause of diarrhoea at this age, leading to dehydration, weight loss, and stunted growth. In cases of untreated infection, coccidiosis can result in the death of the animal [8]. Previous studies [6,7] signalled a significant parasitic problem on Polish alpaca farms. Our research involves a wider representation of farms from different regions of the country, which allows a more detailed determination of the scale and nature of this problem.

Studies carried out in different countries indicate variability in the level of alpaca infection with gastrointestinal parasites, depending on the country and region. In Peru, the extensiveness of alpaca infection with parasites in different regions ranged from 2% to 85% in Cuzco [9]; 41% in Ayacucho, Pampa Galeras [10]; 80.83% in Tacna [11]; and 32.9% in Junín, Paccha [12]. The extensiveness of alpaca infection with parasites in Argentina ranged from 16.04% to 100% in Jujuy [13,14,15,16,17]. The extensiveness of alpaca parasite infection in Bolivia in different regions ranged from 28% to 100% in La Paz, Apolobamba [18]. The study showed marked differences in the extensiveness of *Eimeria* infections in alpacas in different countries. The highest percentage of infected animals was reported in Japan (79.2%) [19] and the Puno region of Peru (87.5%) [20]. Lower infestation rates have been observed in New Zealand (3.2%) [21] and the UK (2.2%) [22], among others. A study conducted at Harbin Zoo (China) in 2015–2016 found that alpacas were infected with gastrointestinal parasites in 38.97% of cases [23]. In Germany and Austria, parasites in alpacas were found in 91% of farms [24].

Our hypothesis is that infection of alpacas (*Vicugna pacos*) with GI parasites varies significantly by region, sex, season, and the age of the animals. As such, there is a need to tailor deworming and prophylaxis programs to the specific needs of individual groups of alpacas. Therefore, the aim of this study was to assess the prevalence and intensity of alpaca (*Vicugna pacos*) infection with GI parasites according to region, sex, season, and age.

## 2. Materials and Methods

### 2.1. Materials

**Figure 1:**
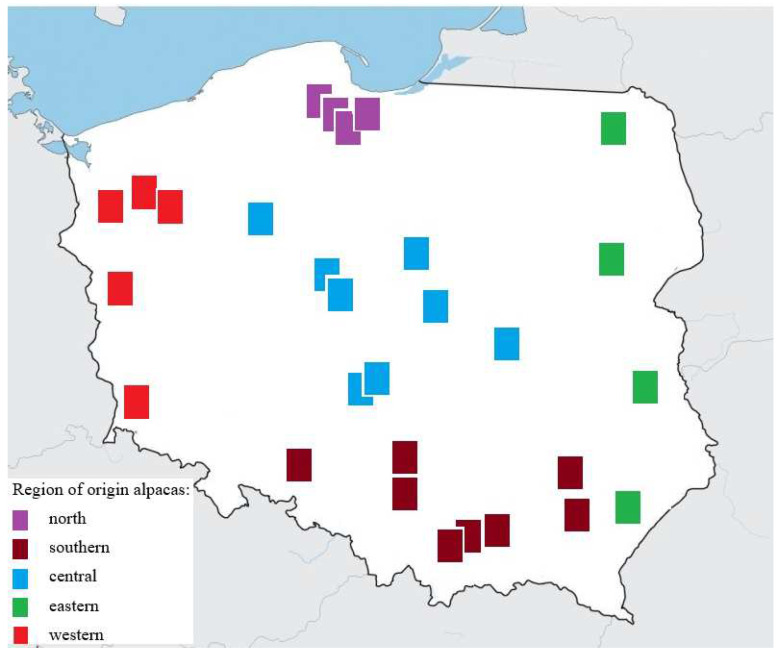
Region of origin alpacas’ herds in Poland.

Alpacas from selected farms across Poland were subjected to parasitological analysis. The examination included a total of 512 animals from 29 herds located throughout Poland. Sampling locations are indicated in Figure 1. The number of individuals examined exceeded the minimum required (*n* = 359), which was calculated on the basis of a sample from the population, assuming a confidence level of 95%, a fraction size of 0.5, and a maximum error of 5%.

Faecal samples were taken once in spring and autumn (September 2023–May 2024) to assess the need for antiparasitic preparations. The owners of the alpacas found no signs of illness, such as diarrhoea, leakage from the eyes or nose, fever, difficulty breathing, or lameness. The animals had a normal appetite, and there were no digestive problems such as bloating or diarrhoea. The alpacas communicated with others in a manner characteristic of their species, showing no aggression, fear, or desire for isolation, suggesting their well-being. They moved freely, were active, and had no mobility problems or weakness. Their external appearance was perfectly normal—they had a healthy coat that was shiny and thick, with no signs of alopecia, scabs, or other skin lesions. All these observations confirmed that the alpacas studied were clinically healthy.

On all farms, the animals were housed in a livestock building during the winter, while during the summer, they were kept in a pasture during the day. The animals on the farms were kept in wooden or brick buildings. Alpacas had access to them all year round. Floors were clay, sand, or concrete. Depending on the size of the herd, the livestock building was divided into sectors, cubicles, and separate passageways. On large farms, there was a division into groups (pregnant females, lactating females with young, weaned young, young females that were over 1.5 years old, young males up to 2 years old, and adult breeding males). The livestock buildings also had herding corridors that allowed easy access to the different sectors. They were also used to drive animals from one part of the building to another. These corridors allowed for the efficient transport of feed and the removal of food residues and faeces.

In summer, the alpacas spent the whole day in fenced pastures. Pastures in large herds of alpacas were used in rotation. This promoted vegetation regeneration and reduced the risk of parasites. Animals were moved to a new pasture when the grass was too short (less than 5 cm). When the pasture was depleted, the alpacas were given hay, which was fed into the feeders rather than directly from the ground. This minimised the risk of parasites. In smaller herds, grazing was carried out on whole pastures. Alpacas excreted faeces in one place in the pasture. Therefore, the pastures were cleaned once a week after the alpacas had spent time there. During hot weather, the alpacas were provided with shade. The animals had unlimited access to clean water. The water was changed regularly, and the water level was constantly replenished. In case of a shortage of grass in the pasture, hay was fed into the feeders.

The alpacas’ diet was mainly based on roughage. Grasses and hay were the basis, and during the winter, they received a supplement of oats, dried beet pulp, or commercial alpaca crisps. During the winter, alpacas on some farms received carrots and dried alfalfa. This supplemented the diet. The alpacas had constant access to fresh water around the clock. All alpacas had access to licks at all times. The animals received a vitamin and mineral supplement for alpacas called Alpamin.

Animals were divided into groups according to sex, age, region of origin, and the time of year in which the study was carried out. Faeces were collected from all alpacas on the farm. The body weight of the animals ranged from 6.5 kg (cria) to 75 kg (adult males). Fifty alpacas were housed in farms located in the north of Poland, 154 in the southern part, 100 in the central part, 177 in the eastern part, and 31 in the western part.

Seasonal variation in infection was also examined: 309 alpacas were tested in spring and 203 in autumn. The animals were kept in separate groups based on age, sex, and physiological status. In addition, as the group comprised both females (*n* = 298) and males (*n* = 214), it was possible to compare parasite infection levels by sex. The age of the alpacas also varied: 119 animals were less than 1 year old (alpacas have immature immune systems, making them more susceptible to parasitic infections), 180 were between 1.5 and 3 years old (the immune system is more developed compared to younger individuals), 103 were between 4 and 6 years old (at this age, they are at full breeding maturity, and their immune system is fully functional), 78 were between 7 and 10 years old (alpacas at this age are still considered adults; their immune system is starting to show signs of weakness, which can lead to greater susceptibility to parasites), and 32 were over 10 years old (the alpacas are in a group where the immune system may be working less effectively, making them more susceptible to parasitic infections; their bodies may find it difficult to cope with parasites).

Based on this data, it was possible to make a detailed assessment of the prevalence and intensity of parasite infection with regard to region, season, sex, and age.

### 2.2. Research Methods

No ethics committee approval was needed for the study. Polish law specifies that agricultural activities related to animal husbandry and breeding, and activities that do not cause animals pain, suffering, distress, or permanent damage to the organism to a degree equal to or more intense than a needle prick do not require ethical approval [25].

Faecal samples were taken directly from the rectum and placed in labelled polythene bags. Samples were stored in a fridge at a temperature of 4 °C until analysis. The date, age, and sex of the animal were recorded at the time of collection.

Coproscopic studies were carried out in two steps. In the first step, preliminary examinations were conducted using the qualitative Willis–Schaaf flotation method (direct flotation), to establish which parasite groups were present in the samples. Two grams of faeces was added to 10 mL of the flotation solution (NaCl, d = 1200 g/mL) and mixed. The suspension was poured into a test tube until a convex meniscus was obtained, and a coverslip was placed on top of the tube. After 10–15 min. a coverslip was removed, placed on a slide, and examined under the microscope [26].

In the second step, the McMaster quantitative method was employed as the main study. Three grams of faeces was placed in a plastic container, and a salt solution (NaCl, d = 1200 g/mL) was added to a final volume of 45 mL. Then, the homogenized sample was sieved, and the filtrate was pipetted into two counting chambers (0.15 mL each). Eggs/oocyst were allowed to float (3–10 min) and then counted. The number of eggs/oocysts in one chamber was multiplied by 100 [26]. The results of the main study are presented in the manuscript.

The prevalence of parasites (eggs/oocysts)(expressed in %), and the intensity of infection (expressed as EPG/OPG—number of eggs/oocysts per 1 g of faeces) were determined [27].

Nematodes were identified according to Taylor et al. [26], Bauer et al. [28], Zajac and Conboy [29], and Thienpont et al. [30]. Parasites were classified as strongylid eggs (including *Haemonchus*, *Ostertagia*, and *Trichostrongylus*), *Nematodirus*, *Strongyloides*, *Eimeria*, and *Trichuris*. *Strongyloides* sp., *Trichuris* sp., and *Nematodirus* spp. eggs were identified to the genus level. Among the nematodes producing eggs with bipolar plugs, *Trichuris* sp. was identified, and the remaining ones were classified as cappilarid type.

Protists of the genus *Eimeria* were identified to the species level according to Bauer et al. [31] and Gomez-Puerta et al. [8]. The oocysts of *Eimeria* vary considerably between species, allowing them to be identified without the need for sporulation [2].

### 2.3. Statistical Analysis

The results were analysed using Statistica 13.3. Since the obtained data did not have a normal distribution (Shapiro–Wilk test), nonparametric tests were used to assess statistically significant differences between the studied variables. The prevalence of individual parasite species was compared using the χ^2^ test, while the intensity of infection was compared using the Mann–Whitney U test and the Kruskal–Wallis test, as appropriate. The confidence interval of a proportion was calculated by the modified Wald method, as recommended by Agresti and Coull [32].

## 3. Results

The mean prevalence of gastrointestinal (GI) parasites was 74.4%. More specifically, 68.8% of the tested animals demonstrated GI nematode infection, and 34.8% demonstrated *Eimeria* infection. The most prevalent GI nematode and protist were *Nematodirus* spp. and *Eimeria punoensis*, respectively (Table 1).

Significant differences were found in the prevalence of some parasites between the studied regions (Table 2). Alpacas from northern Poland demonstrated a significantly lower prevalence of *Strongyloides* sp., Strongylida, and *E. punoensis* compared to other regions. The highest prevalence of *Strongyloides* sp. was recorded in western and southern Poland, while Strongylida and *E. punoensis* were most prevalent in eastern Poland. Alpacas from western Poland were also characterised by a significantly higher prevalence of total *Eimeria* infection. The highest combined infection rate was noted in eastern Poland and the lowest in northern and central Poland.

Significantly higher *Strongyloides* sp. intensity of infection was noted in western Poland than in eastern Poland (Table 2).

Regarding the effect of season, a significantly higher prevalence of *E. macusaniensis* and total *Eimeria* was noted in autumn compared to spring. In addition, *Trichuris* sp. and total nematodes were present in significantly higher numbers in autumn, but *E. macusaniensis*, and total *Eimeria* were more prevalent in spring (Table 3).

It was found that *Eimeria macusaniensis* and *E. lamae* were more prevalent in males than females, as were combined *Eimeria*. However, no significant differences in the intensity of infection were noted between males and females by individual parasite species (Table 4).

It was noted that animals younger than one year of age demonstrated a significantly higher prevalence of infection with regard to *Nematodirus* spp., *Strongyloides* sp., Strongylida, *E. macusaniensis*, *E. lamae*, *E. alpacae*, and *E. punoensis*, as well as total nematodes and *Eimeria*. Juveniles younger than one year of age also demonstrated a higher prevalence of all parasites (Table 5).

In addition, animals younger than one year demonstrated a significantly greater intensity of infection with *Nematodirus* spp., *Trichuris* sp., *E. macusaniensis*, *E. lamae*, *E. alpacae*, and *E. punoensis*, as well as combined nematodes and *Eimeria*. However, they also presented a significantly lower intensity of Strongylida infection compared to other age groups. This group also demonstrated a significantly higher total intensity of parasite infection (Table 5).

The analysis showed differential co-occurrence of various parasite species in alpacas (Table 6). The most common type of infection was mixed infections, in which one host was infected simultaneously by two or more species. Capillarid-type nematodes, *Nematodirus* sp., *Strongyloides* sp., and Strongylida most often co-occurred with one or two other species. However, in the case of some nematodes (e.g., *Nematodirus* sp.), infections involving up to five different parasite species were also observed. Protists of the genus *Eimeria* showed a clear tendency to form complex parasitic systems, often co-occurring with three or four other species. It can be assumed that these parasites are well-adapted to coexist with other species in one host. In general, the most common type of infection was co-infection of two parasite species. Complex parasitic systems were particularly characteristic of *Eimeria* (Table 6).

## 4. Discussion

In the present study, the mean prevalence of overall gastrointestinal (GI) parasites was 74.4%. Almost 68.8% of the alpacas were infected with nematodes, and the most commonly observed was *Nematodirus* spp. The prevalence of this nematode was 38.5%. In Germany, *Nematodirus* spp. was detected in 19.3% of the alpaca samples, similar to Japan, where this nematode was found in 13.2% of samples [24]. In other studies conducted in Poland, *Nematodirus* spp. was found in 33.9% of alpacas [33].

A group of parasitic nematodes commonly found in alpacas are strongylids [7,24,34]. Rashid et al. [34] reported that the prevalence of strongylid eggs (including *Haemonchus*, *Trichostrongylus,* and *Camelostrongylus*) in alpacas from Australia was 47–81%. In Germany, the prevalence of strogylids (excluding *Namatodirus*) was 32,3 to 67%, depending on the region [24], and in Japan, it was 50.9% [19]. In our study, eggs of this group of nematodes were found in ca. 35% of tested alpacas. Similar results were obtained by Nosal et al. [7] in other Polish alpaca herds (40%).

Alpacas can be infected with *Aonchotheca* sp. and other capillarids, which produce eggs with asymmetric bipolar plugs. This study showed that Capillaria-type nematodes had a relatively low prevalence of 8.2%. Similar results were obtained by Hyuga and Matsumoto (5.7%) [19]. Quite large differences in results are observed in the occurrence of *Trichuris* sp. in alpacas. In our study, this nematode was found in 11%; in Australia, it was found in 6% [35], but in Germany, it was found in over 70% of animals [24]. In a study by Pyziel-Serafin et al. [33], also in Poland, the prevalence of *Trichuris* sp. was estimated at 11.7%.

A significantly lower prevalence of *Strongyloides* sp., Strongylid, and *E. punoensis* in alpacas was recorded in northern Poland. The highest prevalence of *Strongyloides* sp. was found in alpacas from western and southern Poland, while Strongylid and *E. punoensis* were most prevalent in the eastern region. In addition, alpacas from western Poland demonstrated a significantly higher intensity of *Strongyloides* sp. infection compared to those from the eastern region. These differences may be due to local environmental conditions and breeding practices, as confirmed by studies from other countries. A study by Hyuga and Matsumoto [19] in Japan indicated that differences in the prevalence of parasitic infections were strongly related to local environmental conditions.

Infections caused by parasites of the genus *Eimeria* pose a serious threat to camelid health, often leading to high mortality [2]. In the present study, four species of *Eimeria* parasites were found: *E. macusaniensis*, *E. lamae*, *E. alpacae*, and *E. punoensis*. Of these, *E. macusaniensis* is considered to be the most pathogenic [2]. Our present findings indicate a higher prevalence of *Eimeria* spp. during the autumn season, which may be due to a number of factors. In particular, seasonal changes in the availability and quality of feed may affect the condition of the alpacas, which may weaken their immune system and increase susceptibility to parasitic infections [34]. Also, the autumn climate, particularly its increased humidity, favours the development and survival of *Eimeria* oocysts in the environment. Increased humidity can accelerate the parasite development cycle, leading to higher numbers on pastures. Also, the alpacas are often restricted to contaminated buildings or pastures from spring to autumn, resulting in prolonged exposure to oocysts and thus an increased risk of parasite infection during the autumn season. This is consistent with the observations of Diaz et al. [35], who noted a relationship between feed availability, weather conditions and animal health. The findings indicate the complexity of the mechanisms affecting the spread of parasites in alpacas with regard to climate-based changes in the environment.

Our data indicate a significantly higher prevalence of *Nematodirus* spp., *Strongyloides* sp., Strongylida, *E. macusaniensis*, *E. lamae*, *E. alpacae,* and *E. punoensis*, as well as nematodes in general and *Eimeria* spp., in juveniles up to one year of age. This finding is in line with those of numerous studies, which indicate that young animals are more susceptible to parasitic infections [36,37,38].

Alpacas can be infected with protists of the genus *Eimeria* on the day of birth, as the minimum prepatent period is 10 days [2]. As young alpacas lack the fully developed defence mechanisms needed to effectively combat parasites, they are more vulnerable to the rapid development and multiplication of parasites. In addition, young alpacas are usually more susceptible to the stress of weaning and dietary changes, which can further weaken their ability to mount an immune response. Furthermore, they often lack the previous contact with parasites needed to develop specific immunity; as such, these initial infections tend to be more intense than those noted in adult animals, which may have developed immunity. Rodríguez et al. [20] found that the highest intensity of *Eimeria* spp. infection was observed in observed in alpacas aged 45–60 days (34.731 OPG). In our studies, the highest intensity of this parasite infection was recorded in <1-year-old animals. As such, younger individuals require special care and prophylaxis to reduce the risk of parasite infections, especially those with high infection intensity. Such measures include regular monitoring of health status, faecal testing for parasites, and the use of deworming programs tailored to young animals; these are all effective methods for controlling parasite numbers in alpaca herds, which has a direct impact on their health.

In the present study, 29.1% of tested alpacas were infected with at least two species of parasites, indicating the complexity of parasitic infections in these animals. The co-occurrence of parasites can affect the health of the host, resulting in a higher burden than infection with a single species. Such infections can lead to increased nutrient loss and poorer health and thus poorer animal performance and welfare [2,39]. The interactions between co-occurring parasites can also amplify their detrimental effects on the host, with one weakening the immune system and increasing susceptibility to other parasites. In addition, parasites can secrete substances that inhibit the host immune response, which again promotes the survival and growth of other parasite species [40]. Multispecies parasitic infections also complicate effective control and treatment because they require varying strategies for selecting antiparasitic agents [41,42], with some parasites being more resistant to standard treatments. Therefore, only regular faecal examinations, optimized grazing rotations, and tailored deworming programs allow effective parasite control and ensure the health of the host animal.

Our findings also show that male alpacas are significantly more infected with *Eimeria macusaniensis* and *Eimeria lamae*, as well as by total *Eimeria*, than females, suggesting they may have higher susceptibility to parasitic infections. This significantly higher infection rate in males may be due to the influence of sex hormones on immunity and specific behaviours that increase the risk of exposure to parasite oocysts; for example, males may exhibit more territorial behaviour and forage more extensively in contaminated pastures, which increases their contact with infected faces and substrate [43]. Additionally, in some cases, males may be more vulnerable to stress related to herd hierarchy. Stress has a direct impact on the functioning of the immune system, leading to a weakening of the immune system [44]. Although little information on the influence of sex on the prevalence of *E. macusaniensis* and *E. lamae* in alpacas, similar phenomena have been widely reported in other animal species. Studies on ruminants, such as sheep, also indicate that males are often more susceptible to parasitic infections, which is explained by both behavioural and physiological differences [45]. For example, in male sheep, it has been observed that greater intensity of infection is associated with territorial competition and more intensive use of pasture, which increases contact with infected oocysts [43,44,46,47].

Comparing the intensity of parasitic infection in alpacas in this study with the results obtained by Gomez-Puerta et al. [8], there are significant differences in the levels of *Eimeria* infection. In our study, the mean intensity of *Eimeria* infection was 324 OPG (range: 50–6250 OPG) with the highest values for *E. punoensis* (365 OPG; range: 50–6000 OPG). For individual *Eimeria* species, the following mean values were recorded: for *E. macusaniensis*—71 OPG, for *E. lamae*—117 OPG, for *E. alpacae*—120 OPG, and for *E. punoensis*—365 OPG. In the study by Gomez-Puerta et al. [8], the mean intensity of *Eimeria* infection in clinically asymptomatic alpacas was 43,920 OPG (range: 100–5,440,000 OPG). Particularly high infection intensity was observed for *E. lamae*, whose mean intensity of infection was 206,600 OPG (range: 100–5,440,000 OPG), which is significantly higher than in our study. The next *Eimeria* species in the study by Gomez-Puerta et al. [8] also showed higher intensity of infection values: *E. alpacae*—4450 OPG, *E. macusaniensis*—4410 OPG, *E. punoensis*—3780 OPG, and *E. ivitaensis*—380 OPG. A higher mean intensity of *Eimeria* infection in alpacas from Peru was observed also by Rodríguez et al. [20]—mean: 24,017 OPG. In turn, the intensity of *Eimeria* infection in this study was higher than the values reported by Nosal et al. [7] in alpacas from Poland. These authors found that the mean intensity of *Eimeria* oocyst output was 213 OPG, while in our study, it was 324 OPG. It should be assumed that the differences noted between our results and those of other authors most likely result from different environmental conditions, including those related to the animals’ housing conditions and meteorological conditions, i.e., air temperature and humidity.

Our present data indicate seasonal differences in the intensity of infection with individual parasites, with a significantly higher intensity of infection noted for *Trichuris* sp. and total nematodes in the autumn season. This may be due to favourable environmental conditions, such as humidity and temperature. Despite lower temperatures during this period, increased humidity may prolong the survival period of invasive forms, increasing the risk of infection. In contrast, a higher intensity of infection by *Eimeria macusaniensis* and total *Eimeria* was reported during spring, which again may be due to higher humidity, higher temperatures, and an increase in the number of oocysts in the environment. These conditions favour the development and survival of oocysts, which become more readily available to juveniles during this period [44].

The differences in the prevalence and intensity of parasite infection noted between different regions of Poland may be due to local environmental factors, breeding management methods, and pasture availability. For example, a significantly lower extensity of infection was found for *Strongyloides* sp. and *Eimeria punoensis* in alpacas from northern Poland. These differences highlight the need for differential parasite prevention and treatment strategies to effectively manage the health of alpacas in different regions [1,48].

A limitation of this study was the use of nonparametric statistical tests to analyse the obtained data and only determining whether there were significant differences between the analysed variables (age, sex, region). However, in subsequent studies, a larger number of samples should be obtained in each of the analysed groups/variables, and a multivariate analysis (e.g., logistic regression) should be applied. Such data analysis would allow for the calculation of odds ratios for different categories, the demonstration of potential relationships between variables, and the indication of how certain factors or combinations of factors affect the occurrence of parasites in different regions.

## 5. Conclusions

This study showed alpacas in Poland to be susceptible to gastrointestinal parasite infection (74.4%), which indicates the need for effective preventive measures. Parasites such as strongylids and *E. punoensis* can cause serious health problems as they reach a high intensity of infection. Significant regional differences were also observed in the prevalence of parasites, particularly *Strongyloides* sp. and strongylids, suggesting that local environmental conditions and husbandry practices have a significant impact on the development of infections. Furthermore, seasonal variations were noted, especially an increase in *Eimeria* infections in autumn.

Parasitic infections in alpacas in Poland are common and generally asymptomatic. In order to effectively prevent the development of parasitic diseases in these animals, it is necessary to regularly conduct parasitological tests, especially in seasons when the risk of infection is the highest. Prevention and treatment programs should be adapted to the conditions in different regions of Poland. The most basic recommendations should be to maintain housing hygiene and perform targeted deworming and rotation of pastures in order to reduce the concentration of parasite eggs, oocysts, and larvae in the environment, which will effectively reduce the risk of infection of animals.

## Figures and Tables

**Table 1:** Prevalence of gastrointestinal parasites and intensity of infection in alpacas.

Parasite	Number of Alpacas Infected	Prevalence (%) (95% CI)	Intensity of Infection (EPG/OPG)		
			Mean	Median	Range
Capillarid-type	42	8.2 (6.1–10.9)	51	50	50–100
*Trichuris* sp.	90	17.6 (14.5–21.1)	73	50	50–200
Strongylida	178	34.8 (30.8–39.0)	342	150	50–2300
*Strongyloides* sp.	164	32.0 (28.1–36.2)	80	50	50–850
*Nematodirus* spp.	197	38.5 (34.4–42.8)	102	50	50–500
Total nematodes	352	68.8 (64.6–72.6)	292	150	50–2300
*E. macusaniensis*	81	15.8 (12.9–19.2)	71	50	50–250
*E. lamae*	68	13.3 (10.6–16.5)	117	50	50–400
*E. alpacae*	83	16.2 (13.3–19.7)	120	50	50–600
*E. punoensis*	93	18.2 (15.1–21.8)	365	150	50–6000
Total *Eimeria*	178	34.8 (30.8–39.0)	324	150	50–6250
Overall total (Nematodes + *Eimeria*)	381	74.4 (70.5–78.0)	422	250	50–6550

**Table 2:** Prevalence of gastrointestinal parasites and intensity of infection in alpacas by region.

Parasite	Area of Poland	N/n	Prevalence (%) (95% CI)	χ^2^ Test Value	Intensity of Infection (EPG/OPG)			
					Mean	Median	Range	Kruskal–Wallis Test Value
Capillarid-type	north	4/50	8.0 (2.6–19.4)	χ^2^ = 0.94; *p* = 0.92	50	50	50–50	H = 3.2; *p* = 0.52
	south	10/154	6.5 (3.4–11.7)		55	50	50–100	
	central	9/100	9.0 (4.6–16.4)		50	50	50–50	
	east	16/177	9.0 (5.6–14.3)		50	50	50–50	
	west	3/31	9.7 (2.6–25.7)		50	50	50–50	
*Trichuris* sp.	north	5/50	10.0 (3.9–21.8)	χ^2^ = 5.34; *p* = 0.25	60	50	50–100	H = 2.2; *p* = 0.69
	south	22/154	14.3 (9.6–20.8)		82	50	50–200	
	central	21/100	21.0 (14.1–30.1)		74	50	50–150	
	east	37/177	20.9 (15.5–27.5)		70	50	50–150	
	west	5/31	16.1 (6.6–33.1)		70	50	50–150	
Strongylida	north	8/50	16.0 (8.1–28.8)	χ^2^ = 23.6; *p* < 0.001	481	250	50–1950	H = 5.3; *p* = 0.26
	south	52/154	33.8 (26.8–41.6)		328	100	50–2100	
	central	25/100	25.0 (17.5–34.7)		398	150	50–1450	
	east	83/177	46.9 (39.7–54.2)		300	100	50–2300	
	west	10/31	32.3 (18.5–50.0)		520	550	50–1500	
*Strongyloides* sp.	north	2/50	4.0 (0.03–14.2)	χ^2^ = 25.8; *p* < 0.001	50	50	50–50	H = 9.7; *p* = 0.05
	south	63/154	40.9 (33.4–48.8)		106 ^ab^	50	50–850	
	central	28/100	28.0 (20.1–37.5)		68 ^ab^	50	50–150	
	east	58/177	32.8 (26.3–40.0)		60 ^a^	50	50–200	
	west	13/31	41.9 (26.4–59.3)		77 ^b^	100	50–100	
*Nematodirus* spp.	north	20/50	40.0 (27.6–53.8)	χ^2^ = 2.59; *p* = 0.63	108	75	50–250	H = 8.3; *p* = 0.08
	south	61/154	39.6 (32.2–14.5)		109	50	50–500	
	central	32/100	32.0 (23.7–41.7)		116	50	50–300	
	east	73/177	41.2 (34.3–68.6)		82	50	50–350	
	west	11/31	35.5 (21.1–53.1)		145	50	50–400	
Combined nematodes	north	29/50	58.0 (44.2–70.6)	χ^2^ = 8.66; *p* = 0.07	228	100	50–2000	H = 3.4; *p* = 0.50
	south	103/154	66.9 (59.1–73.8)		317	150	50–2200	
	central	64/100	64.0 (54.2–72.7)		274	150	50–1600	
	east	135/177	76.3 (69.5–82.0)		279	150	50–2300	
	west	21/31	67.7 (50.0–81.5)		395	200	50–1650	
*E. macusaniensis*	north	8/50	16.0 (8.1–28.8)	χ^2^ = 1.89; *p* = 0.76	56	50	50–100	H = 2.5; *p* = 0.64
	south	23/154	14.9 (10.1–21.5)		61	50	50–100	
	central	18/100	18.0 (11.6–26.8)		86	50	50–250	
	east	25/177	14.1 (9.7–20.1)		75	50	50–200	
	west	7/31	22.6 (11.1–40.1)		64	50	50–100	
*E. lamae*	north	9/50	18.0 (9.5–31.0)	χ^2^ = 3.71; *p* = 0.45	100	50	50–250	H = 0.41; *p* = 0.98
	south	18/154	11.7 (7.4–17.8)		106	50	50–250	
	central	9/100	9.0 (4.6–16.4)		117	100	50–250	
	east	27/177	15.3 (10.7–21.3)		133	50	50–400	
	west	5/31	16.1 (6.6–33.1)		100	50	50–200	
*E. alpacae*	north	8/50	16.0 (8.1–28.8)	χ^2^ = 6.39; *p* = 0.17	94	50	50–350	H = 4.1; *p* = 0.39
	south	23/154	14.9 (10.1–21.5)		141	100	50–350	
	central	11/100	11.0 (6.1–18.8)		127	50	50–450	
	east	32/177	18.1 (13.1–24.5)		105	50	50–550	
	west	9/31	29.0 (15.9–46.8)		133	50	50–600	
*E. punoensis*	north	4/50	8.0 (2.6–19.4)	χ^2^ = 9.44; *p* = 0.05	488	475	50–950	H = 3.4; *p* = 0.49
	south	26/154	16.9 (11.7–23.6)		298	150	50–1100	
	central	14/100	14.0 (8.4–22.3)		732	275	50–6000	
	east	42/177	23.7 (18.0–30.5)		258	100	50–1200	
	west	7/31	22.6 (11.1–40.1)		450	450	50–1250	
Combined *Eimeria*	north	19/50	38.0 (25.8–51.9)	χ^2^ = 9.34; *p* = 0.05	213	100	50–1100	H = 8.6; *p* = 0.07
	south	52/154	33.8 (26.8–41.6)		275	150	50–1400	
	central	26/100	26.0 (18.4–35.4)		548	250	50–6250	
	east	64/177	36.2 (29.4–43.5)		309	175	50–1350	
	west	17/31	54.8 (37.8–70.9)		312	50	50–1500	
Combined total	north	32/50	64.0 (50.1–75.9)	χ^2^ = 12.04; *p* = 0.02	333	175	50–2050	H = 3.0; *p* = 0.56
	south	110/154	71.4 (63.8–78.0)		426	250	50–2200	
	central	69/100	69.0 (59.4–77.3)		461	250	50–6550	
	east	147/177	83.1 (76.8–87.9)		390	250	50–2300	
	west	23/31	74.2 (56.5–86.5)		591	300	50–1950	

N—number of alpacas infected; n—number of alpacas tested; ^a,b^—different letters indicate statistically significant differences at *p* < 0.05.

**Table 3:** Prevalence of gastrointestinal parasites and intensity of infection in alpacas according to season.

Parasite	Season	N/n	Prevalence (%) (95% CI)	χ^2^ Test Value	Intensity of Infection (EPG/OPG)			
					Mean	Median	Range	Mann–Whitney U-TEST Value
Capillarid-type	spring	23/309	7.4 (5.0–11.0)	χ^2^ = 0.60; *p* = 0.44	50	50	50–50	Z = 1.05 *p* = 0.29
	autumn	19/203	9.4 (6.0–14.2)		53	50	50–100	
*Trichuris* sp.	spring	60/309	19.4 (15.4–24.2)	χ^2^ = 1.82; *p* = 0.18	67 ^a^	50	50–150	Z = 2.28 *p* = 0.02
	autumn	30/203	14.8 (10.5–20.4)		87 ^b^	50	50–200	
Strongylida	spring	100/309	32.4 (27.4–37.8)	χ^2^ = 1.98; *p* = 0.16	302	100	50–2300	Z = 1.08 *p* = 0.28
	autumn	78/203	38.4 (32.0–45.3)		395	225	50–2100	
*Strongyloides* sp.	spring	97/309	31.4 (26.5–36.8)	χ^2^ = 0.15; *p* = 0.70	63	50	50–200	Z = 1.45 *p* = 0.15
	autumn	67/203	33.0 (26.9–39.7)		105	50	50–850	
*Nematodirus* spp.	spring	111/309	35.9 (30.8–41.4)	χ^2^ = 2.15; *p* = 0.14	101	50	50–500	Z = −0.10 *p* = 0.92
	autumn	86/203	42.4 (35.8–49.2)		103	50	50–400	
Combined nematodes	spring	212/309	68.6 (63.2–73.5)	χ^2^ = 0.01; *p* = 0.93	247 ^a^	150	50–2300	Z = 2.11 *p* = 0.03
	autumn	140/203	69.0 (62.3–74.2)		359 ^b^	200	50–2200	
*E. macusaniensis*	spring	40/309	12.9 (9.6–17.2)	χ^2^ = 4.84; *p* = 0.03	80 ^a^	50	50–250	Z = −2.06 *p* = 0.04
	autumn	41/203	20.2 (15.2–26.3)		61 ^b^	50	50–150	
*E. lamae*	spring	40/309	12.9 (9.6–17.2)	χ^2^ = 0.08; *p* = 0.78	121	50	50–400	Z = −0.03 *p* = 0.98
	autumn	28/203	13.8 (9.7–19.3)		111	50	50–300	
*E. alpacae*	spring	44/309	14.2 (10.8–18.6)	χ^2^ = 2.23; *p* = 0.14	139	50	50–600	Z = −1.42 *p* = 0.15
	autumn	39/203	19.2 (14.4–25.2)		99	50	50–350	
*E. punoensis*	spring	58/309	18.8 (14.8–23.5)	χ^2^ = 0.19; *p* = 0.66	390	175	50–6000	Z = 0.23 *p* = 0.82
	autumn	35/203	17.2 (12.6–23.1)		324	100	50–1200	
Combined *Eimeria*	spring	92/309	29.8 (24.9–35.1)	χ^2^ = 8.56; *p* = 0.003	401 ^a^	200	50–6250	Z = −3.55 *p* < 0.001
	autumn	86/203	42.4 (35.8–49.2)		242 ^b^	100	50–1400	
Combined total	spring	229/309	74.1 (68.9–78.7)	χ^2^ = 0.04; *p* = 0.85	390	250	50–6550	Z = 1.60 *p* = 0.11
	autumn	152/203	74.9 (68.5–80.4)		467	276	50–2200	

N—number of alpacas infected; n—number of alpacas tested; ^a,b^—different letters indicate statistically significant differences at *p* < 0.05.

**Table 4:** Prevalence of gastrointestinal parasites and intensity of infection in alpacas according to sex.

Parasite	Sex	N/n	Prevalence (%) (95% CI)	χ^2^ Test Value	Intensity of Infection (EPG/OPG)			
					Mean	Median	Range	Mann–Whitney U-Test Value
Capillarid-type	female	26/298	8.7 (6.0–15.5)	χ^2^ = 0.26; *p* = 0.61	50	50	50–50	Z = 1.23 *p* = 0.22
	male	16/214	7.5 (4.6–11.9)		53	50	50–100	
*Trichuris* sp.	female	52/298	17.5 (13.5–22.2)	χ^2^ = 0.01; *p* = 0.93	70	50	50–200	Z = 1.31 *p* = 0.19
	male	38/214	17.8 (13.2–23.5)		78	50	50–150	
Strongylida	female	101/298	33.9 (28.8–39.5)	χ^2^ = 0.24; *p* = 0.62	351	150	50–2100	Z = −0.62 *p* = 0.54
	male	77/214	36.0 (29.9–42.6)		331	150	50–2300	
*Strongyloides* sp.	female	100/298	33.6 (28.4–39.1)	χ^2^ = 0.76; *p* = 0.38	79	50	50–850	Z = 1.56 *p* = 0.12
	male	64/214	29.9 (24.2–36.4)		83	50	50–500	
*Nematodirus* spp.	female	108/298	36.2 (31.0–41.9)	χ^2^ = 1.50; *p* = 0.22	100	50	50–500	Z = 0.81 *p* = 0.42
	male	89/214	41.6 (35.2–48.3)		104	50	50–400	
Combined nematodes	female	202/298	67.8 (62.3–72.8)	χ^2^ = 0.31; *p* = 0.58	292	150	50–2150	Z = 0.70 *p* = 0.48
	male	150/214	70.1 (63.6–75.8)		291	150	50–2300	
*E. macusaniensis*	female	39/298	13.1 (9.7–17.4)	χ^2^ = 4.00; *p* = 0.04	69	50	50–200	Z = −0.31 *p* = 0.76
	male	42/214	19.6 (4.6–11.9)		73	50	50–250	
*E. lamae*	female	29/298	9.7 (6.8–13.7)	χ^2^ = 7.80; *p* = 0.005	124	50	50–400	Z = −0.27 *p* = 0.79
	male	39/214	18.2 (13.6–24.0)		112	50	50–300	
*E. alpacae*	female	49/298	16.4 (12.7–21.1)	χ^2^ = 0.03; *p* = 0.87	115	50	50–600	Z = 0.16 *p* = 0.88
	male	34/214	15.9 (11.6–21.4)		126	50	50–450	
*E. punoensis*	female	48/298	16.1 (12.1–20.8)	χ^2^ = 2.03; *p* = 0.15	257	125	50–1200	Z = 1.80 *p* = 0.07
	male	45/214	21.0 (16.1–27.0)		480	200	50–6000	
Combined *Eimeria*	female	92/298	30.9 (25.9–36.3)	χ^2^ = 4.76; *p* = 0.03	265	125	50–1350	Z = 0.95 *p* = 0.34
	male	86/214	40.2 (33.8–46.9)		387	175	50–6250	
Combined total	female	219/298	73.5 (68.2–78.2)	χ^2^ = 0.32; *p* = 0.57	381	200	50–2150	Z = 1.50 *p* = 0.13
	male	162/214	75.7 (69.5–81.0)		475	300	50–6550	

N—number of alpacas infected; n—number of alpacas tested.

**Table 5:** Prevalence of gastrointestinal parasites and intensity of infection in alpacas according to age.

Parasite	Age (Years)	N/n	Prevalence (%) (95% CI)	χ^2^ Test Value	Intensity of Infection (EPG/OPG)			
					Mean	Median	Range	Kruskal–Wallis Test Value
Capillarid-type	≤1	10/119	8.4 (4.5–14.9)	χ^2^ = 9.00; *p* = 0.06	55	50	50–100	H = 3.20; *p* = 0.52
	1.5–3	21/180	11.7 (7.7–17.2)		50	50	50–50	
	4–6	9/103	8.7 (4.5–16.0)		50	50	50–50	
	7–10	1/78	1.3 (<0.01–7.6)		50	50	50–50	
	>10	1/32	3.1 (<0.01–17.1)		50	50	50–50	
*Trichuris* sp.	≤1	28/119	23.5 (16.8–32.0)	χ^2^ = 6.30; *p* = 0.18	113 ^a^	125	50–200	H = 40.03; *p* < 0.001
	1.5–3	34/180	18.9 (13.8–25.3)		56 ^b^	50	50–150	
	4–6	14/103	13.6 (8.2–21.7)		61 ^b^	50	50–150	
	7–10	9/78	11.5 (6.0–21.0)		50 ^b^	50	50–50	
	>10	5/32	15.6 (6.4–32.2)		50 ^b^	50	50–50	
Strongylida	≤1	53/119	44.5 (35.9–53.5)	χ^2^ = 10.18; *p* = 0.04	124 ^a^	50	50–800	H = 40.37; *p* < 0.001
	1.5–3	61/180	33.9 (27.4–41.1)		426 ^b^	250	50–2300	
	4–6	25/103	24.3 (17.0–33.4)		358 ^b^	250	50–1300	
	7–10	27/78	34.6 (25.0–45.7)		411 ^b^	200	50–1550	
	>10	12/32	37.5 (22.9–54.8)		696 ^b^	700	50–1950	
*Strongyloides* sp.	≤1	43/119	36.1 (28.1–45.1)	χ^2^ = 9.53; *p* = 0.05	60	50	50–200	H = 5.65; *p* = 0.23
	1.5–3	60/180	33.3 (26.9–40.5)		72	50	50–200	
	4–6	36/103	35.0 (26.4–44.6)		96	50	50–500	
	7–10	22/78	28.2 (19.4–39.1)		118	50	50–850	
	>10	3/32	9.4 (2.5–25.0)		67	50	50–100	
*Nematodirus* spp.	≤1	59/119	49.6 (40.8–58.4)	χ^2^ = 19.29; *p* = 0.001	196 ^a^	200	50–500	H = 107.05; *p* < 0.001
	1.5–3	77/180	42.8 (35.8–50.1)		68 ^b^	50	50–250	
	4–6	24/103	23.3 (16.1–32.4)		60 ^b^	50	50–250	
	7–10	28/78	35.9 (26.1–47.0)		50 ^b^	50	50–50	
	>10	9/32	28.1 (15.4–45.5)		50 ^b^	50	50–50	
Combined nematodes	≤1	95/119	79.8 (71.7–86.1)	χ^2^ = 19.58; *p* < 0.001	257 ^a^	200	50–1100	H = 13.78; *p* = 0.008
	1.5–3	132/180	73.3 (66.4–79.3)		292 ^b^	150	50–2300	
	4–6	58/103	56.3 (46.7–65.5)		261 ^b^	100	50–1450	
	7–10	49/78	62.8 (51.7–72.7)		318 ^b^	100	50–1650	
	>10	18/32	56.3 (39.3–71.9)		517 ^ab^	175	50–2000	
*E. macusaniensis*	≤1	45/119	37.8 (29.6–46.8)	χ^2^ = 57.31; *p* < 0.001	86 ^a^	50	50–250	H = 16.30; *p* = 0.003
	1.5–3	20/180	11.1 (7.2–16.6)		53 ^b^	50	50–100	
	4–6	8/103	7.8 (3.8–14.8)		50 ^b^	50	50–50	
	7–10	6/78	7.7 (3.3–16.1)		57 ^b^	50	50–100	
	>10	2/32	6.3 (0.7–21.2)		50 ^b^	50	50–50	
*E. lamae*	≤1	36/119	30.3 (22.7–39.0)	χ^2^ = 43.08; *p* < 0.001	169 ^a^	150	50–400	H = 27.47; *p* < 0.001
	1.5–3	21/180	11.7 (7.9–17.2)		62 ^b^	50	50–150	
	4–6	7/103	6.8 (3.1–13.6)		50 ^b^	50	50–50	
	7–10	2/78	2.6 (0.2–9.4)		50	50	50–50	
	>10	2/32	6.3 (0.7–21.2)		50	50	50–50	
*E. alpacae*	≤1	40/119	33.6 (25.7–42.5)	χ^2^ = 38.66; *p* < 0.001	178 ^a^	100	50–600	H = 18.50; *p* = 0.001
	1.5–3	25/180	13.9 (9.5–19.8)		56 ^b^	50	50–100	
	4–6	7/103	6.8 (3.1–13.6)		71 ^b^	50	50–150	
	7–10	10/78	12.8 (6.9–22.2)		90 ^b^	50	50–400	
	>10	1/32	3.1 (<0.01–17.1)		50	50	50–50	
*E. punoensis*	≤1	50/119	42.0 (33.5–51.0)	χ^2^ = 67.12; *p* < 0.001	585 ^a^	400	50–6000	H = 38.87; *p* < 0.001
	1.5–3	30/180	16.7 (11.9–22.8)		110 ^b^	50	50–650	
	4–6	8/103	7.8 (3.8–14.8)		100 ^b^	50	50–350	
	7–10	3/78	3.9 (0.9–11.2)		83	50	50–150	
	>10	2/32	6.3 (0.7–21.2)		175	175	50–300	
Combined *Eimeria*	≤1	85/119	71.4 (62.7–78.8)	χ^2^ = 97.77; *p* < 0.001	545 ^a^	350	50–6250	H = 60.33; *p* < 0.001
	1.5–3	54/180	30.0 (23.8–37.1)		131 ^b^	100	50–700	
	4–6	19/103	18.5 (12.1–27.1)		108 ^b^	50	50–400	
	7–10	14/78	18.0 (10.9–28.0)		118 ^b^	100	50–450	
	>10	6/32	18.8 (8.5–35.7)		100 ^b^	50	50–300	
Combined total	≤1	110/119	92.4 (86.1–96.1)	χ^2^ = 39.26; *p* < 0.001	643 ^a^	450	50–6550	H = 59.00; *p* < 0.001
	1.5–3	139/180	77.2 (70.5–82.8)		328 ^b^	200	50–2300	
	4–6	61/103	59.2 (49.6–68.2)		282 ^b^	150	50–1450	
	7–10	51/78	65.4 (54.3–7.0)		338 ^b^	100	50–1700	
	>10	20/32	62.5 (45.2–77.1)		495 ^b^	225	50–2050	

N—number of alpacas infected; n—number of alpacas tested; ^a,b^—different letters indicate statistically significant differences at *p* < 0.05.

**Table 6:** Occurrence of single- and multi-species infections in alpacas.

Parasite	Number of Infected Animals	Type of Infection Number of Infected Animals (Prevalence, %)						
		1-Species	2-Species	3-Species	4-Species	5-Species	6-Species	7-Species
Capillarid-type	42	2 (4.76%)	20 (47.62%)	16 (38.10%)	3 (7.14%)	1 (2.38%)	-	-
*Trichuris* sp.	90	11 (12.22%)	35 (38.89%)	34 (37.78%)	9 (10.00%)	1 (1.11%)	-	-
Strongylida	178	22 (12.36%)	86 (48.31%)	60 (33.71%)	9 (5.06%)	1 (0.56%)	-	-
*Strongyloides* sp.	164	33 (20.12%)	70 (42.68%)	51 (31.10%)	9 (5.49%)	1 (0.61%)	-	-
*Nematodirus* spp.	197	55 (27.92%)	89 (45.18%)	46 (23.35%)	6 (3.05%)	1 (0.51%)	-	-
*E. macusaniensis*	81	15 (18.52%)	42 (51.85%)	23 (28.40%)	1 (1.23%)	-	-	-
*E. lamae*	68	17 (25.00%)	40 (58.82%)	10 (14.71%)	1 (1.47%)	-	-	-
*E. alpacae*	83	15 (18.07%)	47 (56.63%)	20 (24.10%)	1 (1.20%)	-	-	-
*E. punoensis*	93	12 (12.90%)	55 (59.14%)	25 (26.88%)	1 (1.08%)	-	-	-
Capillarid-type	42	2 (4.76%)	12 (28.57%)	13 (30.95%)	6 (14.29%)	6 (14.29%)	3 (7.14%)	0 (0.00%)
*Trichuris* sp.	90	6 (6.67%)	21 (23.33%)	21 (23.33%)	17 (18.89%)	17 (18.89%)	7 (7.78%)	1 (1.11%)
Strongylida	178	14 (7.87%)	53 (29.78%)	38 (21.35%)	36 (20.22%)	29 (16.29%)	7 (3.93%)	1 (0.56%)
*Strongyloides* sp.	164	27 (16.46%)	44 (26.83%)	33 (20.12%)	28 (17.07%)	23 (14.02%)	9 (5.49%)	0 (0.00%)
*Nematodirus* spp.	197	34 (17.26%)	54 (27.41%)	43 (21.83%)	40 (20.30%)	19 (9.64%)	6 (3.05%)	1 (0.51%)
*E. macusaniensis*	81	5 (6.17%)	4 (4.94%)	17 (20.99%)	26 (32.10%)	21 (25.93%)	7 (8.64%)	1 (1.23%)
*E. lamae*	68	5 (7.35%)	15 (22.06%)	15 (22.06%)	15 (22.06%)	14 (20.59%)	3 (4.41%)	1 (1.47%)
*E. alpacae*	83	1 (1.20%)	6 (7.23%)	21 (25.30%)	28 (33.73%)	22 (26.51%)	4 (4.82%)	1 (1.20%)
*E. punoensis*	93	2 (2.15%)	15 (16.13%)	15 (16.13%)	28 (30.11%)	24 (25.81%)	8 (8.60%)	1 (1.08%)
Total	381	96 (25.20)	112 (29.40)	72 (18.90)	56 (14.70)	35 (9.19)	9 (2.36)	1 (0.26)

## Data Availability

The data presented in this study are available upon request from the corresponding author.
